# 

**Published:** 1909-09-15

**Authors:** 


CONTENTS

Event and Comment - - - - - - - - 443
Knowing How, - - - - *By O. M. Matteson, D.D.S.* 445
The Nature of Alloys, - - - *By M. L. Ward, D.D.Sc.* 452
Reflex Pains in the Trifacial Nerve, *By Chalmers J. Lyons, D.D.S.* 461
Tightening Loose Plates, - - *By F. G. Worthley, D.D.S.* 467
Pertaining to Pyorrhea, - - - *By J. D. Patterson, D.D.S.* 470
Humane Methods and Suggestions for Practice,
*By W. G. Ebersole, M.D., D.D.S.* 476
Indents - - - - - - - - - - - 486
Announcements - - - - - - - - - 496

